# Protective Effects of N-Acetylcysteine Against Schizophrenia-Related Behavioral and Parvalbumin Interneuron Deficits Induced by Adolescent Stress

**DOI:** 10.1093/schizbullopen/sgaf029

**Published:** 2025-11-13

**Authors:** Ícaro S Freitas, Francisco S Guimarães, Felipe V Gomes

**Affiliations:** Department of Pharmacology, Ribeirão Preto Medical School, University of São Paulo, Avenida Bandeirantes 3900, Ribeirão Preto, SP, 14049-900, Brazil; Department of Pharmacology, Ribeirão Preto Medical School, University of São Paulo, Avenida Bandeirantes 3900, Ribeirão Preto, SP, 14049-900, Brazil; Department of Pharmacology, Ribeirão Preto Medical School, University of São Paulo, Avenida Bandeirantes 3900, Ribeirão Preto, SP, 14049-900, Brazil

**Keywords:** trauma, adolescence, psychosis, oxidative stress, antioxidants

## Abstract

**Background and Hypothesis:**

Adolescent stress has been linked to an increased risk of developing psychiatric disorders, including schizophrenia. Previous findings from our group suggest that adolescent stress causes redox imbalance and functional impairments in parvalbumin (PV) interneurons and their associated perineuronal nets (PNNs) in the ventral hippocampus (vHip). These changes are associated with behavioral abnormalities, vHip hyperactivity, and dopamine system overdrive, mirroring observations in people with schizophrenia. Thus, we hypothesized that the antioxidant N-acetylcysteine (NAC) could mitigate schizophrenia-related alterations induced by adolescent stress in adult rats.

**Study Design:**

Male Sprague–Dawley rats were subjected to a combination of daily footshock and restraint stress during adolescence [postnatal days (PD) 31-40]. NAC (900 mg/L) was administered through the drinking water either during (PD31-40) or after the adolescent stress (PD51-60). In adulthood (PD63), rats underwent behavioral tests to assess anxiety-like behaviors, social interaction, and cognition. From PD70, *in vivo* recordings of dopamine neurons in the ventral tegmental area (VTA) and immunostaining of PV, PNNs, and the oxidative stress marker 8-hydroxy-2'-deoxyguanosine (8-Oxo-dG) in the vHip were performed.

**Study Results:**

Adolescent stress causes, in adulthood, anxiety-like responses, deficits in sociability and cognitive function, increased VTA dopamine neuron population activity, reduced PV^+^ cells in the vHip, including those surrounded by PNNs, and enhanced expression of 8-Oxo-dG, particularly in PV^+^ cells. NAC treatment, whether administered during or after adolescent stress, significantly attenuated these alterations.

**Conclusions:**

NAC effectively mitigates schizophrenia-related changes induced by adolescent stress and may serve as a pharmacological intervention for prevention and treatment strategies.

## Introduction

Psychiatric disorders have multifactorial origins, arising from an interplay between genetic predispositions and adverse socio-environmental factors.^1^ Among these factors, adverse experiences during childhood and adolescence, critical periods of brain development, are increasingly recognized as significant risk factors.^2^ Exposure to stressors during these vulnerable stages can profoundly affect neurobiological processes, contributing to the development of psychiatric disorders, including schizophrenia.^3^^,^^4^

To elucidate the mechanisms underlying the deleterious effects of adolescent stress, studies from our group have shown that exposing adolescent rats to stressors triggers a cascade of neurobiological changes in the ventral hippocampus (vHip). These include redox imbalance and functional impairments in parvalbumin (PV)-expressing GABAergic interneurons and their associated perineuronal nets (PNNs).^5^ Such alterations are linked to a disruption in the excitatory/inhibitory (E/I) balance in the vHip, resulting in vHip hyperactivity, downstream overactivation of the dopamine system in the ventral tegmental area (VTA), and behavioral deficits.^5–8^ These changes resemble core features observed in schizophrenia, where *hyperdopaminergia* and PV interneuron dysfunction are hallmarks of schizophrenia pathophysiology.^9^^,^^10^

The fact that adolescent stress causes redox dysregulation highlights the potential for antioxidant-based interventions to mitigate stress-induced neurobiological and behavioral impairments related to schizophrenia. Preclinical studies using different animal models for schizophrenia point to increased oxidative stress as a common mechanism underlying deficits in PV interneurons and their associated PNNs.^11^

N-acetylcysteine (NAC), an antioxidant agent with potential use in psychiatry, has demonstrated efficacy for alleviating schizophrenia-related symptoms in preclinical models and clinical trials.^12–15^ Notably, NAC has shown promise in improving cognitive deficits in people with schizophrenia, addressing a core symptom domain of the disorder.^16–18^ However, the potential of NAC to prevent or reverse the long-term effects of adolescent stress has yet to be fully elucidated.

In this study, we investigated the effects of NAC treatment on long-lasting schizophrenia-related neurobiological and behavioral changes induced by adolescent stress in rats. Specifically, we assessed its effects on behavioral impairments, dopamine VTA neurons activity, PV interneurons and PNN deficits, and expression of the marker of DNA damage caused by oxidative stress 8-hydroxy-2'-deoxyguanosine (8-Oxo-dG) in the vHip. Our study aims to advance the understanding of the therapeutic potential of NAC as a preventive or treatment strategy for mitigating schizophrenia-related changes associated with adolescent stress.

## Methods

### Animals

Male Sprague–Dawley rats, aged 21 days (postnatal day 21, PD21), were obtained from the Central Animal Facility of the University of São Paulo, Ribeirão Preto campus. The rats were housed in pairs (2 rats/cage) in their homecages and maintained in a temperature-controlled room (24 ± 2 °C) with a 12/12-h light/dark cycle. Food and water were provided *ad libitum*. All procedures complied with Brazilian and international guidelines for the care and use of laboratory animals. The experiments were approved by the Ethics Committee for Animal Use of Ribeirão Preto Medical School (protocol #155/2018).

### Stress protocol

The adolescent stress exposure protocol was performed as previously described.^6^^,^^19^^,^^20^ Rats during adolescence (PD 31-40) were subject to 25 daily inescapable footshocks (1 mA, 2 s), delivered pseudo-randomized. In addition, the rats underwent restraint stress for 1 h on PD 31, 32, and 40, using a size-adjusted Plexiglas cylindrical restraint tube. Non-stressed (naïve) rats were left undisturbed in their homecages.

Only male rats were used in this study, as previous findings indicated that female adolescent rats exhibited resistance to behavioral and electrophysiological changes following exposure to this adolescent stress protocol.^21^

### NAC treatment

NAC (900 mg/L; Sigma) was administered in the drinking water either during (PD31-40) or after adolescent stress exposure (PD51-60). The dose was based on Zhu et al. (2021),^15^ who showed that NAC at this concentration mitigated impairments in PV and PNN expression, reduced the increased expression of oxidative stress markers (8-Oxo-dG) in the thalamic reticular nucleus, and attenuated the VTA dopamine system hyperactivity in rats exposed to methylazoxymethanol acetate (MAM) *in utero*, a well-established neurodevelopmental model of schizophrenia.^15^ In addition, although we did not measure plasma NAC or glutathione levels, previous preclinical studies have shown that NAC supplementation at similar concentrations in drinking water increases systemic and/or tissue glutathione and exerts antioxidant effects in rodents.^22^

### Behavioral tests

*Light–dark box (LDB) test*—The LDB test is commonly used to evaluate anxiety-related behaviors in rodents.^23^ The apparatus consists of two compartments, one light and one dark (23x20x28 cm each), connected by a central opening. The behavior of the rats was recorded for 5 min using the AnyMaze software (Stoelting), and the time spent in the light compartment was analyzed.

#### Social interaction

The social interaction test was performed as previously described.^24^ The rats were placed in a circular arena (60 cm in diameter and 65 cm in height) for a 10-min habituation period. Immediately after habituation, an unfamiliar rat (social stimulus) of the same species, age, and sex was placed into the arena. Social interaction time was recorded as the time the experimental animal actively interacted with the social stimulus, exhibiting behaviors such as following, grooming, sniffing, or climbing on/under the unfamiliar rat.

#### Novel object recognition test

The novel object recognition (NOR) test was performed as previously described.^24^ Rats were first habituated in a circular arena (60 cm in diameter and 65 cm in height) for 10 min. Twenty-four hours later, they were placed in the arena containing two identical objects for 5 min (acquisition trial). One hour after the acquisition trial, the rats were returned to the arena, where one of the familiar objects was replaced with a novel object. The rats were allowed to explore freely the objects for 5 min (retention trial). The exploration time of the objects was recorded in both trials, considering behaviors such as sniffing, licking, observing, or touching the objects with the front paws. A discrimination index was calculated as follows: discrimination index = (Time spent exploring novel object − Time spent exploring familiar object) / (Time spent exploring novel object + Time spent exploring familiar object).

### In vivo electrophysiological activity of VTA dopamine neurons

Animals were anesthetized with 8% chloral hydrate (400 mg/kg, i.p), and stereotaxic surgery was performed using the following coordinates: 5.5–5.7 mm in the anteroposterior from the bregma; 0.6 mm lateral from the midline; and 6.5–9.0 mm ventral from the brain surface. A glass electrode filled with 2% Chicago Sky Blue in 2 M NaCl was introduced into the brain to target the VTA. Recordings of dopamine neurons were conducted for 1 to 3 min across six to nine tracks, spaced 0.2 mm apart, in a pre-determined pattern.^6^ Dopamine neurons were identified based on previously established criteria described.^25^

The parameters analyzed included the number of spontaneously active dopamine neurons per track (population activity), mean firing rate, and the percentage of spikes occurring in burst. Additionally, these parameters were evaluated according to VTA subregions (medial, central, and lateral). Data regarding firing frequency and the percentage of firings occurring in bursts were acquired using the Neuroexplorer software (NexTechnologies).

### Immunofluorescence for PV, PNNs, and the oxidative stress marker 8-Oxo-dG in the vHip

Animals not subjected to electrophysiology were perfused for subsequent immunofluorescence to investigate the long-term impact of adolescent stress on the expression of PV, PNN, and 8-Oxo-dG in the vHip as previously described.^5^ Rats were deeply anesthetized with 25% urethane (1 mL/100 g) and transcardially perfused with 0.01 M phosphate-buffered saline, followed by 4% paraformaldehyde. Coronal brain sections were collected in the rostrocaudal direction, targeting the vHip, with each section having a thickness of 30 μm. The tissues were incubated for 24 h at 4 °C with constant agitation in a solution containing rabbit anti-PV antibody (1:2000, Swant, #PV25); biotinylated *Wisteria floribunda* agglutinin (WFA; 1:1000, VectorLabs, #B1355) for PNN labeling, and mouse anti-8-Oxo-dG antibody (1:500, Abcam, #ab6262). After incubation, the tissues were washed and incubated for 90 min with constant agitation in a solution containing the following secondary antibodies: goat anti-rabbit AlexaFluor® 594 (1:1000, Invitrogen, #A11037); AlexaFluor® 488 conjugated with streptavidin (1:1000, Invitrogen, #S11223), and goat anti-mouse AlexaFluor® 647 (1:1000, Abcam, #ab150115). Five to eight sections per animal were washed and mounted on silanized slides with Fluoroshield mounting medium with DAPI (Abcam, #ab104139) to visualize the vHip region, specifically the terminal part of the ventral CA1 and ventral subiculum. Images were acquired using a Leica® SP8 confocal microscope with an interface to the Leica Application Suite software (Leica Systems®). The counting of PV^+^ and PNN^+^ cells and the fluorescence intensity of PV, PNN, and 8-Oxo-dG were analyzed using the Fiji software (NIH). Fluorescence intensity for each PV^+^ cell was obtained by calculating the Corrected Total Cell Fluorescence (CTCF), expressed in arbitrary units (a.u.), following previously described methods with adaptations.^26^ The CTFC was determined considering the integrated density (IntDen), the area of the selected cell's soma (AC), and the mean background fluorescence (MBF), according to the formula: CTCF = IntDen – (AC) × (MBF).

### Experimental design

**Figure 1:**
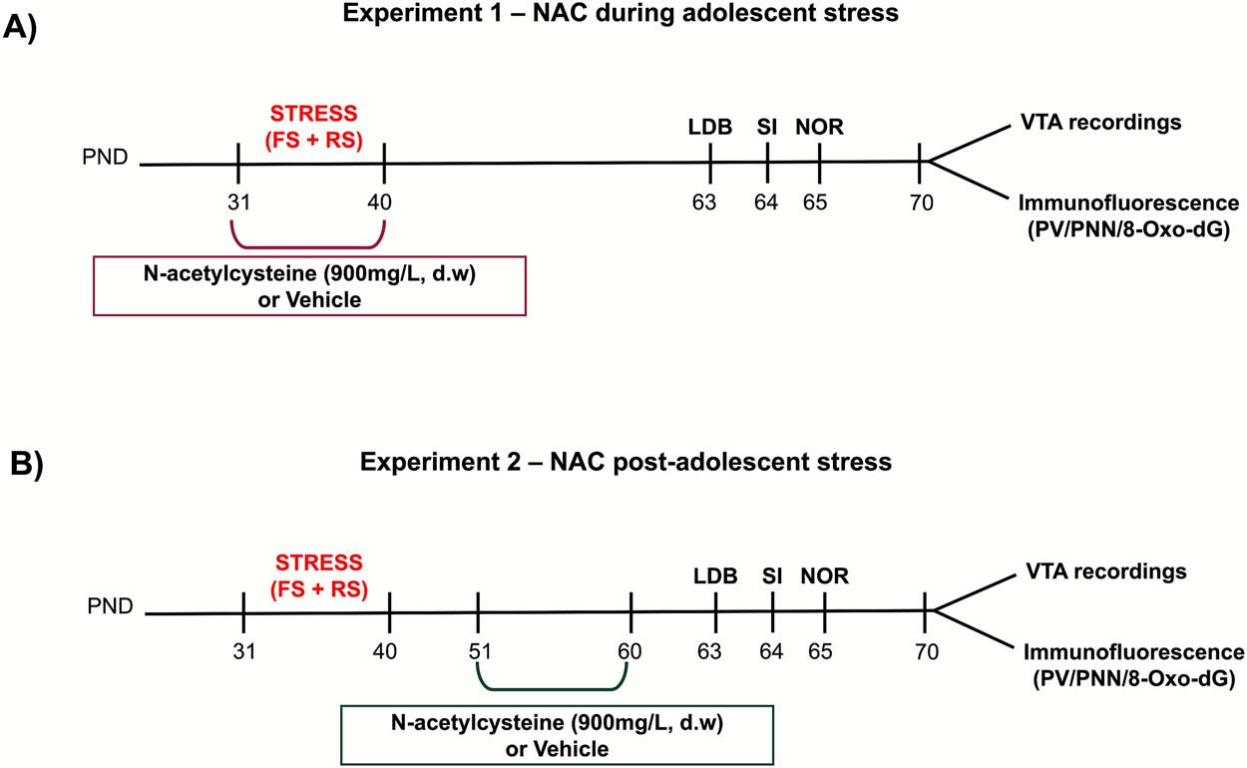
Experimental design. (A) Experiment 1. Rats were exposed to a combination of stressors—Footshock (FS) and restraint stress (RS)—From postnatal day (PND) 31 to 40. During this period, they received either vehicle or N-acetylcysteine (900 mg/L) in drinking water (d.w.). Beginning on PND 63 (adulthood), behavioral tests were conducted to assess anxiety-like behavior [light–dark box (LDB) test], sociability [social interaction (SI)], and cognitive function [novel object recognition (NOR) test]. On PND 70, animals underwent either *in vivo* electrophysiological recordings of VTA dopamine neurons or brain perfusion for immunofluorescence to assess PV, PNN, and 8-Oxo-dG expression in the vHip. (B) Experiment 2. Rats were subjected to the same stress protocol (FS + RS) between PND 31 and 40. Ten days after the end of stress exposure, they received either vehicle or N-acetylcysteine (900 mg/L) in drinking water (d.w.) through PND 51 and 60. In adulthood (PND 63), the same behavioral tests were performed. From PND 70 onwards, animals underwent either *in vivo* electrophysiological recordings of VTA dopamine neurons or brain perfusion for immunofluorescence.

Two experiments were conducted to evaluate the effects of NAC treatment on the deleterious impact of adolescent stress in adult animals. In the first experiment (Figure 1A), adolescent rats were exposed to inescapable footshocks and restraint stress (PD 31–40). NAC treatment was administered throughout the stress protocol. In adulthood (PD 63–65), the animals underwent behavioral testing, including the LDB, social interaction, and NOR tests. On PD70, one cohort of these animals was used for *in vivo* electrophysiological recordings of VTA dopamine neuron activity. In contrast, another cohort had their brains perfused for immunofluorescence staining for PV, PNNs, and 8-Oxo-dG in the vHip. In the second experiment (Figure 1B), adolescent rats were subjected to the same stress protocol (PD 31–40). Ten days after the stress protocol ended, the animals received NAC treatment for 10 days (PD 51–60). Behavioral tests (LDB, social interaction, and NOR tests) were conducted between PD63 and PD65. On PD70, one cohort underwent VTA electrophysiological recordings, and another had their brains perfused for immunofluorescence analysis.

### Statistical analyses

All data were tested for homogeneity of variances (Bartlett's Test) and normality (Shapiro–Wilk Test). Data were categorized as parametric or non-parametric accordingly. Parametric data were analyzed using 2- or 3-way analysis of variance (ANOVA), followed by Tukey's post-hoc test. Non-parametric data were analyzed using the Kruskal-Wallis test, followed by Dunn's post-hoc test. Statistical significance was defined as *P* < .05.

## Results

### NAC treatment either during or after adolescent stress mitigated the stress-induced behavioral changes in adulthood

The combination of footshock and restraint stress applied to adolescent rats led to long-lasting anxiety-like responses, deficits in social interaction, and impaired object discrimination memory in adulthood. NAC treatment during and post-adolescent stress mitigated all these behavioral abnormalities, except for the anxiety-like responses, which were only attenuated by NAC treatment during the stress period. Importantly, since NAC was administered through drinking water, we assessed water intake and found no changes caused by stress exposure or NAC treatment (Supplementary Figure 1).

**Figure 2:**
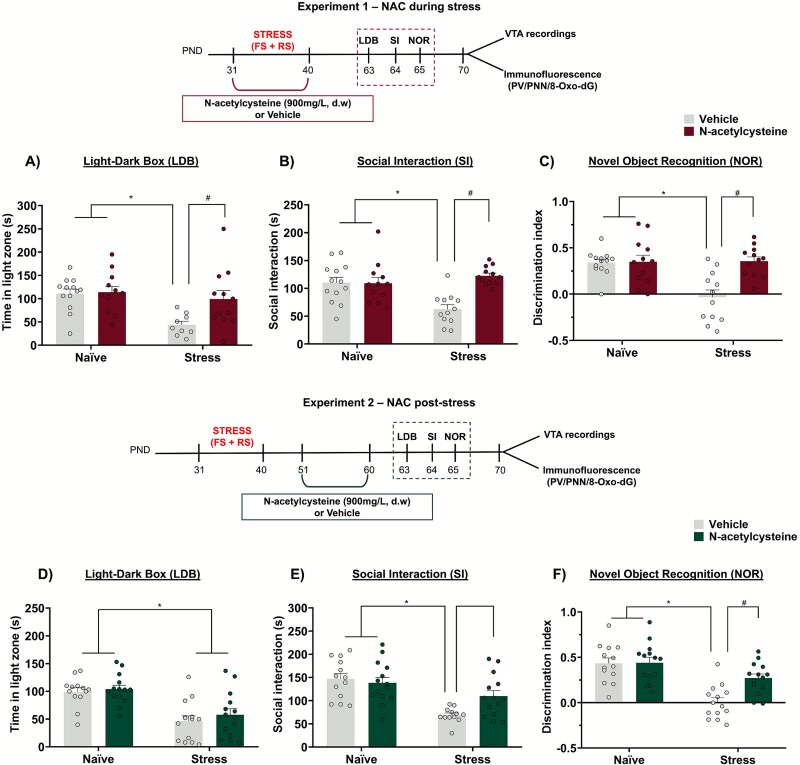
Effects of NAC treatment on behavioral changes induced by adolescent stress in adult rats (n = 12–14/group). NAC treatment during adolescent stress attenuated stress-induced anxiety-like responses and deficits in sociability and cognition indicated, respectively, by (A) decreased exploration of the light zone of the LDB, (B) lower social interaction, and (C) impaired novel object recognition. (D–F) similar findings were observed with the NAC treatment post-adolescent stress, except for the LDB, where NAC post-stress was ineffective. ^*^P < .05, naïve + vehicle/naïve + NAC vs. stress + vehicle; ^#^  *P* < .05, stress + vehicle vs. stress + NAC; 2-way ANOVA followed by Tukey’s post-test. Outliers identified by the ROUT regression test (Q = 1%) were excluded from the analyses in the CCE tests of experiment 1 (n = 2 from stress + vehicle) and experiment 2 (n = 2 from stress + vehicle).

In the experiments assessing the effects of NAC treatment during adolescent stress, for anxiety-like responses in the LDB test, a 2-way ANOVA revealed an interaction between stress and treatment (F_1,42_ = 3.79, *P* = .048) on the time spent in the light compartment. Stressed animals spent less time in the light compartment (*P* < .05 vs. naïve + vehicle, Tukey’s post-test), indicating an anxiety-like behavior. NAC treatment during stress prevented this change (*P* < .05 vs. stress + vehicle, Tukey’s post-test; Figure 2A). For social interaction, an interaction between stress and treatment (F_1,45_ = 12.17, *P* = .001) was also observed. Tukey's post-test revealed that stressed animals spent less time engaging in social interaction (*P* < .05 vs. naïve + vehicle), which was prevented by NAC (*P* < .05 vs. stress + vehicle; Figure 2B). In the NOR test, a 2-way ANOVA also indicated an interaction between stress and treatment (F_1,45=_10, *P* = .003) on the discrimination index. Stressed animals exhibited a lower object discrimination index (*P* < .05 vs. naïve + vehicle, Tukey’s post-test), indicating a cognitive impairment. NAC treatment during stress also prevented this deficit (*P* < .05 vs. stress + vehicle, Tukey’s post-test; Figure 2C).

In the experiments investigating the effects of NAC treatment initiated 10 days after adolescent stress, for anxiety-like responses in the LDB test, a 2-way ANOVA indicated an effect of stress (F_1,51_ = 30.18, *P* < .0001), without an effect of treatment or interaction between stress and treatment. Stressed animals spent less time in the light compartment regardless of treatment (*P* < .05 vs. naïve groups, Tukey’s post-test; Figure 2D), indicating that NAC treatment post-stress did not mitigate the stress-induced anxiety-like responses in adulthood. For social interaction, a 2-way ANOVA indicated an interaction between stress and treatment (F_1,49=_5.14, *P* = .02). Tukey's post-test showed that stressed animals spent less time in social interaction (*P* < .05 vs. naïve + vehicle), which was mitigated by NAC (*P* = .05 vs. stress + vehicle; Figure 2E). For the discrimination index of the NOR test, there was an interaction between stress and treatment (F_1,51_ = 5.98, *P* = .02). Tukey's post-test revealed that stress impaired object recognition memory (*P* < .05 vs. naïve + vehicle, Tukey’s post-test), an effect that was also mitigated by NAC treatment post-stress (*P* < .05 vs. stress + vehicle, Tukey’s post-test; Figure 2F).

### NAC treatment during and post-stress mitigated the enhanced VTA dopamine system activity caused by adolescent stress

Adolescent stress enhanced VTA dopamine system activity in adulthood, indicated by an increased number of spontaneously active VTA dopamine neurons observed through *in vivo* electrophysiology. NAC, administered either during or after adolescent stress, mitigated this enhancement.

**Figure 3:**
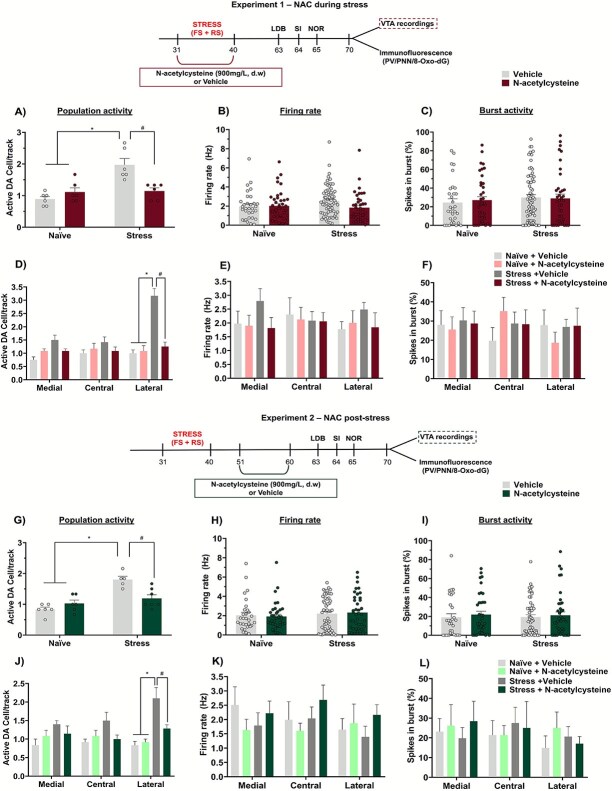
Effects of NAC treatment on the activity of the VTA dopamine system of adult rats subject to adolescent stress (n = 5–7/group). (A) Animals exposed to adolescent stress presented a higher number of spontaneously active VTA dopamine neurons, which was attenuated by NAC treatment during the stress. No change was found in (B) firing rate and (C) burst activity. (D) Further analysis indicated that the enhanced VTA dopamine neuron population activity caused by adolescent stress was confined to the lateral VTA, again without changing (E) firing rate and (F) burst activity [NAC during stress: naïve + vehicle: n = 32 cells; naïve + NAC: n = 40 cells; stress + vehicle: n = 71 cells; stress + NAC: n = 41 cells]. Similar findings on (G and J) VTA dopamine neuron population activity, (H and K) firing rate and (I and L) burst activity were observed when NAC was administered post-adolescent stress [NAC post-stress: Naïve + vehicle: n = 30 cells; naïve + NAC: n = 37 cells; stress + vehicle: n = 65 cells; stress + NAC: n = 43 cells]. ^*^P < .05, naïve + vehicle/naïve + NAC vs. stress + vehicle, ^#^  *P* < .05, stress + vehicle vs. stress + NAC; 2-way ANOVA followed by Tukey’s post-test.

In the experiments investigating the effects of NAC treatment during adolescent stress, a 2-way ANOVA revealed an interaction between stress and treatment (F_1,20_ = 14.80, *P* = .001) on VTA dopamine neuron population activity. Tukey's post-test indicated that stressed animals had a higher number of spontaneously active VTA dopamine neurons (*P* < .05 vs. naïve + vehicle), a change that was prevented by NAC (*P* < .05 vs. stress + vehicle; Figure 3A). Firing rate and percentage of spikes in bursts of DA cells did not differ significantly across all groups (Figure 3B and C).

Further analyses showed that the increased VTA dopamine neuron population activity caused by adolescent stress was confined to the lateral VTA. A 3-way ANOVA indicated an interaction between stress, treatment, and VTA subregion (F_2,60_ = 5.55, *P* = .006). Tukey’s post-test revealed an increase in the number of spontaneously active dopamine neurons specifically in the lateral VTA of stressed animals (*P* < .05 vs. naïve + vehicle, Tukey’s post-test), which was prevented by NAC (*P* < .05 vs. stress + vehicle, Tukey’s post-test; Figure 3D). There were no significant changes in either average firing rate or percentage of spikes in burst at any VTA subregion across groups (Figure 3E and F).

Similar findings were observed with NAC treatment post-stress. A 2-way ANOVA revealed an interaction between stress and treatment (F_1,20_ = 14.20, *P* = .001) on the number of spontaneously active VTA dopamine neurons. Stressed animals exhibited a higher number of spontaneously active VTA dopamine neurons (*P* < .05 vs. naïve + vehicle, Tukey’s post-test), which was mitigated by NAC (*P* < .05 vs. stress + vehicle; Figure 3G). No change was found in firing rate and burst activity (Figure 3H and I). Once again, stressed animals showed increased dopamine neuron population activity confined to the lateral VTA (*P* < .05 vs. naïve + vehicle, Tukey’s post-test), which was mitigated by NAC post-stress (*P* < .05 vs. stress + vehicle, Tukey’s post-test; Figure 3J), with no significant changes in firing rate or burst activity at any VTA subregion across groups (Figure 3K and L).

### NAC treatment, whether administered during or after adolescent stress mitigated deficits in PV^+^ cells, including those surrounded by PNNs, and reduced the expression of the oxidative stress marker 8-Oxo-dG in the vHip of adult rats

We previously found that adolescent stress decreased the number of PV^+^ cells, including PV^+^ cells wrapped by PNNs, and increased the expression of 8-Oxo-dG, particularly in PV^+^ cells, in the vHip.^5^ Our current results expand these findings by indicating that NAC treatment, during or after adolescent stress, mitigates these changes.

**Figure 4:**
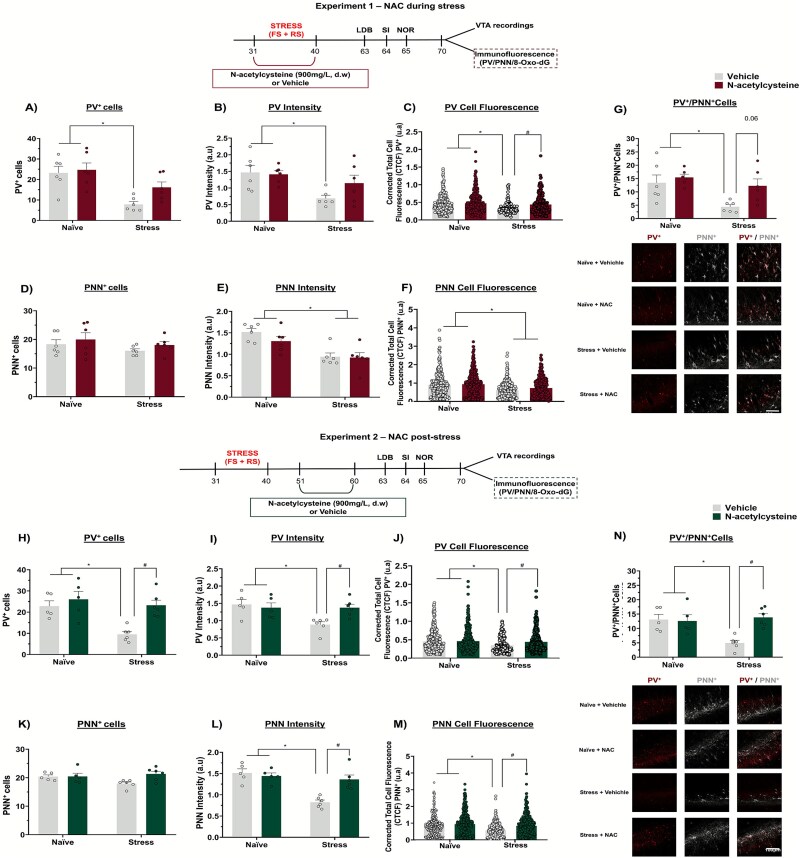
Effects of NAC treatment on PV/PNN in the vHip of adult rats subjected to stress in adolescence (n = 5–6 rats/group). Adolescent stress decreased (A) the number of PV^+^ cells, as well as (B) fluorescence intensity and (C) CTFC of PV^+^ cells compared controls (naïve). Animals treated with NAC during stress did not show these impairments. (D) No change was found in the number of PNN^+^ cells. However, adolescent stress decreased (E) PNN intensity and (F) CTFC of PNN^+^ cells. NAC did not prevent these changes. Furthermore, (G) a reduction in PV^+^/PNN^+^ cells in the vHip of adult rats was observed as a result of exposure to adolescent stress. NAC treatment during stress attenuated this change (NAC during stress: PV – CTFC; naïve + vehicle: n = 274 cells; naïve + NAC: n = 390 cells; stress + vehicle: n = 110 cells; stress + NAC: n = 246 cells. PNN – CTFC; naïve + vehicle: n = 327 cells; naïve + NAC: n = 327 cells; stress + vehicle: n = 244 cells; stress + NAC: n = 322 cells). Similar results were observed with the NAC treatment 10 days after stress (H-K), except that in this protocol NAC reversed the decreases in (L) PNN intensity and (M) CTFC of PNN^+^ cells. Similar to the treatment during stress, (N) NAC post-stress reversed the reduction in PV^+^/PNN^+^ cells in the vHip caused by adolescent stress. [NAC post-stress: PV – CTFC; naïve + vehicle: n = 323 cells; naïve + NAC: n = 335 cells; stress + vehicle: n = 135 cells; stress + NAC: n = 294 cells. PNN – CTFC; naïve + vehicle: n = 303 cells; naïve + NAC: n = 304 cells; stress + vehicle: n = 320 cells; stress + NAC: n = 390 cells]. Scale bar = 100 μm. ^*^P < .05, naïve + vehicle/naïve + NAC vs. stress + vehicle; ^#^*P* < .05, stress + vehicle vs. stress + NAC; 2-way ANOVA followed by Tukey's post-test.

Regarding the number of PV^+^ cells, in the experiments investigating the effects of NAC treatment during adolescent stress, a 2-way ANOVA revealed an interaction between stress and treatment (F_1,20_ = 4.88, *P* = .03). Stressed animals showed a lower number of PV^+^ cells in the vHip (*P* < .05 vs. naïve + vehicle, Tukey’s post-test). This deficit was not observed in stressed animals treated with NAC during the stress protocol (*P* > .05 vs. naïve + vehicle, Tukey’s post-test; Figure 4A). Exposure to stress reduced PV intensity compared to the control group (*P* < .05 vs. naïve + vehicle, Tukey’s post-test). However, this impairment was not observed in animals stressed during adolescence but received NAC treatment during the protocol (*P* > .05 vs. naïve + vehicle, Tukey’s post-test, Figure 4B). By analyzing the individual fluorescence of each PV^+^ cell (CTCF) among the groups, the Kruskal-Wallis test revealed a significant effect on PV intensity (H = 28.41, *P* < .0001). Dunn's post-test indicated that the PV^+^ cells in the vHip of stressed animals had lower fluorescence intensity (*P* < .05 vs. naïve + vehicle, Dunn's post-test), indicating a decreased PV expression in stressed animals' vHip. NAC treatment during stress prevented these changes (*P* < .05 vs. stress + vehicle, Dunn's post-test; Figure 4C).

No difference in the number of PNN^+^ cells was observed between the groups (Figure 4D). However, exposure to stress during adolescence led to a reduction in fluorescence intensity (*P* < .05 vs. naïve + vehicle, Tukey’s post-test, Figure 4E) and CTCF (*P* < .05 vs. naïve + vehicle, Dunn's post-test) of PNN^+^ cells. Treatment with NAC during stress failed to attenuate these changes (*P* < .05 vs. naïve + vehicle, Tukey’s post-test, Figure 4F).

NAC treatment during stress prevented the adolescent stress-induced deficits in the number of PV^+^ cells surrounded by PNNs (PV^+^/PNN^+^ cells). A 2-way ANOVA revealed effects of stress (F_1,20_ = 8.40, *P* = .008) and treatment (F_1,20_ = 5.60, *P* = .02), but no interaction. Tukey's post-test indicated that stressed rats had fewer PV^+^/PNN^+^ cells (*P* < .05 vs. naïve + vehicle), a change prevented by NAC (*P* = .06 vs. stress + vehicle; Figure 4G).

Similar findings were observed with NAC treatment initiated 10 days after adolescent stress exposure. Concerning PV^+^ cells, a 2-way ANOVA revealed an interaction between stress and treatment (F_1,18=_4.26, *P* = .05). Stressed animals presented a lower number of PV^+^ cells in the vHip (*P* < .05 vs. naïve + vehicle, Tukey’s post-test), which was mitigated by NAC post-stress (*P* < .05 vs. stress + vehicle, Tukey’s post-test; Figure 4H). Furthermore, adolescent stress exposure decreased PV fluorescence intensity (*P* < .05 vs. naïve + vehicle, Tukey’s post-test, Figure 4I) and CTFC (*P* < .05 vs. naïve + vehicle, Dunn's post-test, Figure 4J) relative to non-stressed rats, and NAC treatment reduced these impairments on vHip.

As with NAC treatment during the stress protocol, no differences were observed between the groups in the number of PNN^+^ cells (Figure 4K). However, the stressed group showed a reduction in PNN intensity (*P* < .05 vs. naïve + vehicle, Tukey’s post-test, Figure 4L) and CTCF of PNN (*P* < .05 vs. naïve + vehicle, Dunn's post-test; Figure 4M) compared to the control group. Notably, NAC treatment after stress decreased these impairments. Also, NAC post-stress mitigated the impairment for PV^+^/PNN^+^ cells induced by adolescent stress protocol. A 2-way ANOVA revealed an interaction between stress and treatment (F_1,18_ = 9.03, *P* = .007). Tukey's post-test indicated that stressed rats had fewer PV^+^/PNN^+^ cells (*P* < .05 vs. naïve + vehicle), which was attenuated by NAC treatment (*P* < .05 vs. stress + vehicle; Figure 4N).

**Figure 5:**
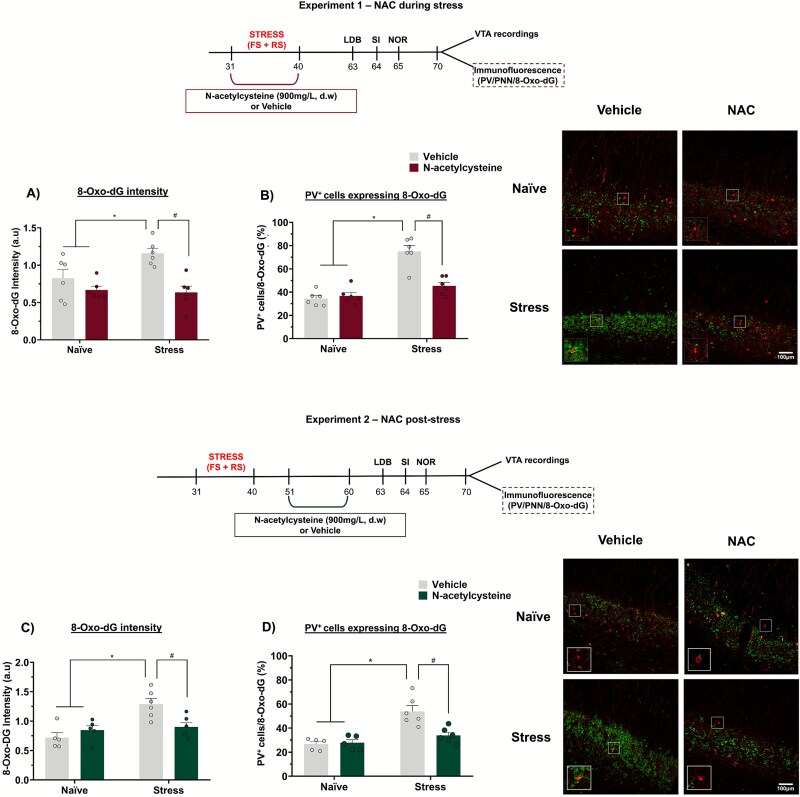
Effects of NAC treatment during or after adolescent stress on 8-Oxo-dG expression in the vHip (n = 5-6/group). (A) Adolescent stress increased 8-Oxo-dG intensity and (B) the percentage of PV^+^ cells expressing 8-Oxo-dG in the vHip. NAC treatment during stress prevented these changes. (C and D) similar findings were observed with NAC administered 10 days after the end of the adolescent stress. Scale bar = 100 μm. ^*^P < .05 naïve + vehicle/naïve + NAC vs. stress + vehicle; #*P* < .05, stress + vehicle vs. stress + NAC; 2-way ANOVA followed by Tukey’s post-test.

Regarding the expression of 8-Oxo-dG, in the experiments investigating the effects of NAC during adolescent stress, a 2-way ANOVA indicated an interaction between stress and treatment (F_1,20_ = 4.88, *P* = .03) on the intensity of 8-Oxo-dG labeling. Stressed rats presented a higher fluorescence intensity of 8-Oxo-dG (*P* < .05 vs. naïve + vehicle, Tukey's post-test), which was prevented by the treatment with NAC during stress (*P* < .05 vs. stress + vehicle, Tukey’s post-test, Figure 5A). Additionally, we assessed the percentage of PV^+^ cells expressing 8-Oxo-dG. A 2-way ANOVA revealed an interaction between stress and treatment (F_1,20_ = 20.75, *P* = .0002). Stressed animals had a greater proportion of PV^+^ cells expressing 8-Oxo-dG (*P* < .05 vs. naïve + vehicle, Tukey’s post-test), which was also prevented by NAC during stress (*P* < .05 vs. stress + vehicle, Tukey’s post-test, Figure 5B). Similar findings were observed for the effects of NAC post-stress. A 2-way ANOVA indicated an interaction between stress and treatment (F_1,18_ = 9.25, *P* = .007) on the intensity of 8-Oxo-dG labeling. Tukey's post-test indicated that stressed rats had a greater 8-Oxo-dG intensity (*P* < .05 vs. naïve + vehicle), which was mitigated by NAC post-stress (*P* < .05 vs. stress + vehicle; Figure 5C). Concerning the percentage of PV^+^ cells expressing 8-Oxo-dG, a 2-way ANOVA revealed an interaction between stress and treatment (F_1,18_ = 9.25, *P* = .007). Tukey's post-test indicated that stressed rats had a greater proportion of PV^+^ co-labelled with 8-Oxo-dG (*P* < .05 vs. naïve + vehicle), which was also mitigated by NAC post-stress (*P* < .05 vs. stress + vehicle; Figure 5D).

## Discussion

As demonstrated in this and other studies,^5^^,^^7^^,^^24^^,^^27^ the application of combined stressors (daily footshock sessions and restraint stress) during the adolescent period in rats (PND 31-40) results in behavioral impairments in adulthood, including anxiety-like behaviors, reduced sociability, and cognition deficits. In addition, adolescent stress exposure leads to increased VTA dopamine system activity, resembling alterations observed in individuals with schizophrenia and animal models for the disorder.^28^^,^^29^ A dysfunction in PV interneurons in brain regions that regulate the VTA dopamine system, such as the vHip, and consequent disruptions in local E/I balance, have been suggested as one of the sources of the VTA dopamine system overdrive. Our results indicate vHip PV/PNN dysfunction following adolescent stress exposure, associated with increased expression of oxidative stress markers such as 8-Oxo-dG in PV+ cells. The treatment with NAC during and after adolescent stress attenuated stress-induced behavioral impairments, VTA dopamine system hyperactivity, PV/PNN alterations, and increased 8-Oxo-dG expression in the vHip of adult rats.

Consistent with findings from other studies by our group,^5^^,^^7^^,^^19^^,^^24^^,^^27^ adolescent stress exposure induced anxiety-like behaviors in adult rats, as indicated by a reduced time exploring the light compartment in the LDB test. Preventive NAC treatment during the adolescent stress protocol mitigated anxiety-like behaviors, whereas NAC treatment initiated 10 days after the stress protocol did not produce the same effect. The mechanisms and neural substrates underlying these differences require further investigation. Evidence suggests that the anxiolytic effects of chronic NAC treatment involve reductions in oxidative stress, neuroinflammation, and modulation of glutamatergic neurotransmission in regions such as the prefrontal cortex, hippocampus, and amygdala.^12^^,^^30^

We also assessed the impact of adolescent stress on social behavior and cognition, using the social interaction and novel object recognition tests, respectively. As reported in our previous studies,^5^^,^^7^^,^^24^^,^^27^ adolescent stress decreased social interaction and impaired short-term object recognition memory; deficits that parallel those seen in animal models for schizophrenia.^31^ NAC treatment administered both during and after the adolescent stress attenuated these social and cognitive deficits. Similarly, preventive NAC treatment in animals subjected to maternal immune activation combined with unpredictable peripubertal stressors (a two-hit schizophrenia model) prevented sociability and cognition impairments.^32^

Beyond behavioral impairments, adolescent stress increased VTA dopamine system activity. Similar findings have been reported by our group using the same stress protocol^5^^,^^7^^,^^19^^,^^24^^,^^27^ and in the MAM neurodevelopmental disruption model for schizophrenia.^28^^,^^33^ In rats stressed during adolescence, dopamine system changes were more prominent in the lateral VTA, which predominantly projects to the associative striatum. In this region, hyperdopaminergic states are more pronounced in schizophrenia.^29^^,^^34^ NAC treatment during and 10 days after stress attenuated the increased number of active dopamine neurons across the VTA, particularly in the lateral subregion. In the MAM model, juvenile NAC treatment (PD11–25) similarly attenuated VTA dopamine system hyperactivity, alongside reductions in 8-Oxo-dG and PV/PNN impairments in the thalamic reticular nucleus.^15^

The overactivation of the dopamine system is a hallmark of schizophrenia. It has been proposed that this heightened dopamine activity is driven by abnormalities in cortical structures, with the vHip playing a key role in modulating VTA alterations. An E/I imbalance in the vHip, primarily due to functional impairments in PV interneurons and their associated PNNs, disrupts the regulation of glutamate pyramidal neuron activity.^35^ Here, we observed reduced PV/PNN expression in the vHip of animals exposed to adolescent stress.

PV interneurons exhibit fast-spiking activity, which can increase reactive species generation.^36^ Functional loss of PV interneurons has been linked to oxidative damage in animal studies.^37^ Their activity requires an effective antioxidant microenvironment largely provided by PNNs.^27^^,^^38^ Both PNNs and PV interneurons in the vHip are not fully mature during adolescence, with maturation concluding in adulthood.^6^^,^^39^ Adverse factors like stress can disrupt this maturation, causing lasting abnormalities.^8^^,^^32^

Despite a reduction in PV+ cells in the vHip of stressed animals, no differences in PNN+ cells between groups were detected, contrasting with other studies using the same adolescent stress protocol.^27^ Possible explanations include: (1) PNNs can regenerate after degradation within days [^40^], indicating PNN restoration post-stress; (2) PNN formation may depend on PV function, and reduced PV expression could impact PNN formation, potentially concentrating extracellular matrix restoration in other neurons.^41^^,^^42^

Regarding PNN fluorescence intensity, adolescent stress reduced labeling intensity, consistent with previous studies.^5^ NAC treatment initiated 10 days post-stress (PND 51-60) mitigated this reduction, whereas treatment during stress did not. The reasons for these differences between treatment windows remain unclear and warrant further investigation. One possibility is that stress-induced PNN loss becomes more pronounced from PND 51 onward,^6^ making NAC treatment during this period more effective at reducing PNN damage.

Post-mortem studies of individuals with schizophrenia reported reduced PV expression in the hippocampus.^9^^,^^43^ Similar changes have been observed in animal models for schizophrenia.^44^ These findings align with reduced GABAergic markers and increased glutamate production in hippocampal regions of patients and high-risk individuals for schizophrenia,^44^^,^^45^ accompanied by anatomical and physiological changes such as increased blood flow and reduced hippocampal volume.^46–48^ In animal models, such as the MAM model, increased vHip activity linked to PV interneuron hypofunction supports the hypothesis that local E/I imbalance underlies schizophrenia-related abnormalities, including VTA dopamine system overdrive.^49^

Oxidative stress is a significant factor in psychiatric disorders, including schizophrenia.^50^ Additionally, redox dysregulation has been proposed as a link between childhood/adolescent stress and a more severe clinical phenotype in early psychosis.^51^ Consistent with previous studies,^5^ we observed increased 8-Oxo-dG expression in the vHip of adult rats exposed to adolescent stress, associated with reduced PV expression, indicating oxidative damage to PV interneurons. NAC treatment during and after stress attenuated reductions in PV/PNN cells and increased 8-Oxo-dG, potentially correlating with the behavioral and electrophysiological improvements. NAC has also been effective in reducing PV/PNN impairments and oxidative stress in cortical structures such as the anterior cingulate cortex in the Gclm knockout (Gclm KO) model,^37^ the thalamic reticular nucleus in the MAM model,^15^ and the prefrontal cortex in neonatal vHip lesion^52^ and NMDA receptor antagonism rodent models.^53^

NAC is widely used as an antioxidant and dietary supplement, with good tolerability and minimal adverse effects. Its primary mechanism involves promoting glutathione synthesis, which underlies its antioxidant properties.^54^^,^^55^ Due to these pharmacotherapeutic characteristics, NAC has attracted considerable interest in clinical and preclinical psychiatric research.^12^^,^^30^^,^^56^ In the present study, NAC mitigated stress effects without inducing alterations in non-stressed (naïve) animals.

Some clinical studies suggest NAC may serve as an adjunctive treatment in schizophrenia, though efficacy appears influenced by treatment duration, individual factors, and disorder stage.^16^^,^^30^^,^^57^^,^^58^ A meta-analysis found that NAC administration for 24 weeks or more significantly improves negative and general symptoms on the Positive and Negative Syndrome Scale, benefiting cognition.^58^ In individuals with early psychosis, NAC treatment remains under investigation. Although the effects on positive and negative symptoms are limited, some studies suggest that NAC may improve cognitive function, especially in working memory and information processing speed.^17^^,^^18^ NAC treatment in this population has been associated with increased cortical glutathione levels^59^^,^^60^ and improved white matter integrity.^18^^,^^57^ Preliminary findings also suggest that NAC may enhance auditory processing and cortical function in recent psychosis.^61^ NAC has also been proposed as a potential preventive intervention in individuals at clinical high risk for psychosis.^62^

Most clinical studies involving NAC have been conducted in adults with recent-onset schizophrenia or psychosis. However, although few studies have investigated whether NAC could serve as a preventive treatment in early adults (18-40 years of age) at clinical high risk for psychosis,^62^ no studies have been conducted in individuals at younger ages. Our study examined whether NAC administration influences its efficacy in mitigating the long-term effects of adolescent stress. The first treatment protocol tested NAC during adolescence to assess its preventive potential, while the second evaluated whether initiating treatment 10 days after the end of the stress could reverse established alterations. Notably, NAC treatment was effective both in preventing and reversing adolescent stress-induced behavioral, electrophysiological, and PV/PNN expression changes in the vHip of rats assessed in adulthood.

Some limitations should be acknowledged. The pharmacological mechanisms by which NAC treatment mitigates PV+ cell impairments induced by adolescent stress were not investigated. We previously reported altered dynamics of reduced and oxidized glutathione following adolescent stress,^5^ and oxidative stress consistently contributes to PV/PNN dysfunction in animal studies.^11^ NAC restores brain glutathione levels and protects PV interneuron function.^37^^,^^63^ Additionally, NAC modulates proteins involved in mitochondrial fusion/fission, such as dynamin-related protein 1, and enhances glutathione peroxidase-1 activity, both of which are essential for mitochondrial integrity and oxidative stress regulation.^64^ Other potential mechanisms, including modulation of astrocyte activity and attenuation of neuroinflammation,^12^ remain to be investigated. In addition, although the behavioral tests used here, such as the LDB, social interaction, and NOR, are commonly used in rodent research and provide valuable insights into anxiety, sociality, and cognition, their direct relevance to the complex symptomatology of schizophrenia in humans is limited. To better model schizophrenia-relevant behaviors, paradigms with higher translational validity, including prepulse inhibition (PPI) of the startle reflex and amphetamine-induced hyperlocomotion, would be more appropriate, as they more closely reflect sensorimotor gating deficits and dopaminergic dysregulation observed in patients. Future studies incorporating these assays could provide deeper insights into schizophrenia-like phenotypes induced by our adolescent stress protocol. In addition, including only male rats in this study represents another important limitation. Some of the assessed outcomes here were not altered in females exposed to the same adolescent stress protocol.^21^ It is important to note that schizophrenia prevalence is nearly equal between sexes, although males typically manifest symptoms earlier than females.^65^ Moreover, sex differences in behavioral and neurobiological impairments are well documented in animal models for schizophrenia.^66^

Overall, our findings suggest that NAC may have translational potential when administered at different phases along the trajectory of stress-related psychopathology. Moreover, they point to NAC as a promising pharmacological strategy for preventing schizophrenia in high-risk individuals, who typically exhibit increased stress responsivity,^67^ and treating schizophrenia symptoms. Further studies are needed to elucidate the mechanisms by which NAC exerts these effects, modifying the disorder’s pathophysiology and indicating novel targets for treatment and prevention.

## Supplementary Material

Supplementary_material_sgaf029
