# Supplementary material for: Protective Effects of N-Acetylcysteine Against Schizophrenia-Related Behavioral and Parvalbumin Interneuron Deficits Induced by Adolescent Stress
Source: Schizophr Bull Open. 2025 Nov 13;6(1):sgaf029. doi: 10.1093/schizbullopen/sgaf029 (PMC12667614; doi:10.1093/schizbullopen/sgaf029)
Supplement: Supplementary_material_sgaf029 [file supplementary_material_sgaf029.docx]

*Supplementary material*

**Protective effects of N-acetylcysteine against schizophrenia-related behavioral and parvalbumin interneuron deficits induced by adolescent stress**

Ícaro S. Freitas, Francisco S. Guimarães, and Felipe V. Gomes

**
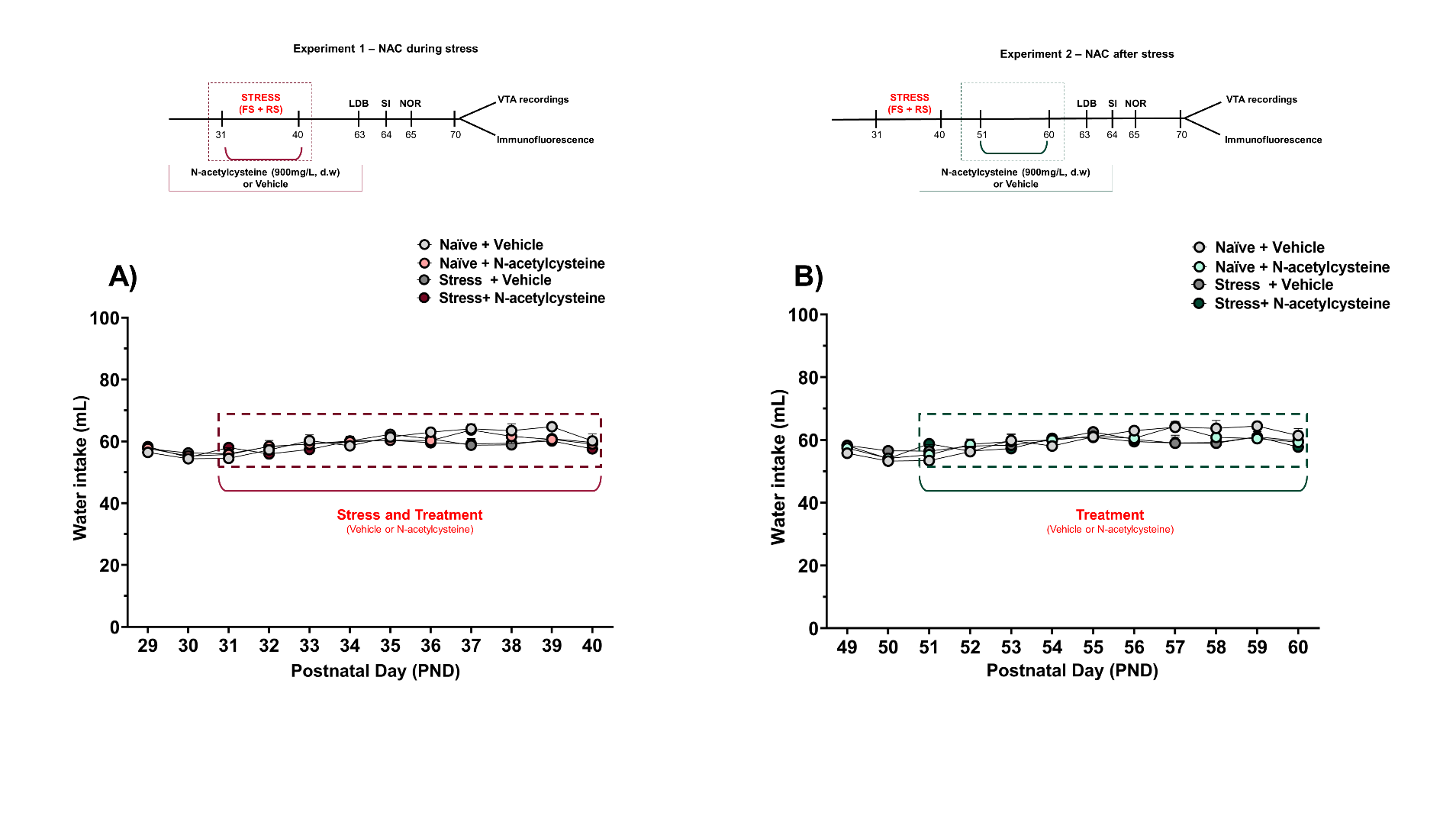
**

**Supplementary figure 1.** Water intake by rats during (A) and ten days after (B) the stress protocol. The water consumption was not affected by exposure to stress (during or long-term) or by the addition of NAC to the drinking water. Intake was measured per homecage (7–8 homecages/ group), with each cage housing two rats.
